# Information Mode–Dependent Success Rates of Obtaining German Medical Informatics Initiative–Compliant Broad Consent in the Emergency Department: Single-Center Prospective Observational Study

**DOI:** 10.2196/65646

**Published:** 2024-12-17

**Authors:** Felix Patricius Hans, Jan Kleinekort, Melanie Boerries, Alexandra Nieters, Gerhard Kindle, Micha Rautenberg, Laura Bühler, Gerda Weiser, Michael Clemens Röttger, Carolin Neufischer, Matthias Kühn, Julius Wehrle, Anna Slagman, Antje Fischer-Rosinsky, Larissa Eienbröker, Frank Hanses, Gisbert Wilhelm Teepe, Hans-Jörg Busch, Leo Benning

**Affiliations:** 1 University Emergency Department Medical Center—University of Freiburg, Faculty of Medicine University of Freiburg Freiburg Germany; 2 Institute of Medical Bioinformatics and Systems Medicine Medical Center—University of Freiburg, Faculty of Medicine University of Freiburg Freiburg Germany; 3 German Cancer Consortium (DKTK) Partner Site Freiburg, A Partnership Between DKFZ and Medical Center University of Freiburg Freiburg Germany; 4 FREEZE-Biobank, Zentrum für Biobanking Medical Center—University of Freiburg, Faculty of Medicine University of Freiburg Freiburg Germany; 5 Institute for Medical Biometry and Statistics Medical Center—University of Freiburg, Faculty of Medicine University of Freiburg Freiburg Germany; 6 Data Integration Center Medical Center—University of Freiburg, Faculty of Medicine University of Freiburg Freiburg im Breisgau Germany; 7 Health Services Research in Emergency and Acute Medicine Charité—Universitätsmedizin Berlin Berlin Germany; 8 Department for Infectious Diseases and Infection Control University Hospital Regensburg Regensburg Germany; 9 University Hospital of Old Age Psychiatry and Psychotherapy University of Bern Bern Switzerland

**Keywords:** biomedical research, delivery of health care, informed consent, medical informatics, digital health, emergency medical services, routinely collected health data, data science, secondary data analysis, data analysis, biomedical, emergency, Germany, Europe, prospective observational study, broad consent, inpatient stay, logistic regression analysis, health care delivery, inpatients

## Abstract

**Background:**

The broad consent (BC) developed by the German Medical Informatics Initiative is a pivotal national strategy for obtaining patient consent to use routinely collected data from electronic health records, insurance companies, contact information, and biomaterials for research. Emergency departments (EDs) are ideal for enrolling diverse patient populations in research activities. Despite regulatory and ethical challenges, obtaining BC from patients in ED with varying demographic, socioeconomic, and disease characteristics presents a promising opportunity to expand the availability of ED data.

**Objective:**

This study aimed to evaluate the success rate of obtaining BC through different consenting approaches in a tertiary ED and to explore factors influencing consent and dropout rates.

**Methods:**

A single-center prospective observational study was conducted in a German tertiary ED from September to December 2022. Every 30th patient was screened for eligibility. Eligible patients were informed via one of three modalities: (1) directly in the ED, (2) during their inpatient stay on the ward, or (3) via telephone after discharge. The primary outcome was the success rate of obtaining BC within 30 days of ED presentation. Secondary outcomes included analyzing potential influences on the success and dropout rates based on patient characteristics, information mode, and the interaction time required for patients to make an informed decision.

**Results:**

Of 11,842 ED visits, 419 patients were screened for BC eligibility, with 151 meeting the inclusion criteria. Of these, 68 (45%) consented to at least 1 BC module, while 24 (15.9%) refused participation. The dropout rate was 39.1% (n=59) and was highest in the telephone-based group (57/109, 52.3%) and lowest in the ED group (1/14, 7.1%). Patients informed face-to-face during their inpatient stay following the ED treatment had the highest consent rate (23/27, 85.2%), while those approached in the ED or by telephone had consent rates of 69.2% (9/13 and 36/52). Logistic regression analysis indicated that longer interaction time significantly improved consent rates (*P*=.03), while female sex was associated with higher dropout rates (*P*=.02). Age, triage category, billing details (inpatient treatment), or diagnosis did not significantly influence the primary outcome (all *P*>.05).

**Conclusions:**

Obtaining BC in an ED environment is feasible, enabling representative inclusion of ED populations. However, discharge from the ED and female sex negatively affected consent rates to the BC. Face-to-face interaction proved most effective, particularly for inpatients, while telephone-based approaches resulted in higher dropout rates despite comparable consent rates to direct consenting in the ED. The findings underscore the importance of tailored consent strategies and maintaining consenting staff in EDs and on the wards to enhance BC information delivery and consent processes for eligible patients.

**Trial Registration:**

German Clinical Trials Register DRKS00028753; https://drks.de/search/de/trial/DRKS00028753

## Introduction

Historically, medical research has been grounded in the systematic observation and detailed description of abnormalities in human anatomy and physiology, which were identified as diseases. The practice of involving humans in research became common due to the need for controlled experiments, which are essential for advancing medical knowledge. However, this progress has been marred by unethical practices, where individuals were subjected to research against their will or without their knowledge, highlighting the need for robust safeguards. Consequently, important regulations have evolved to enable ethically acceptable research on and with humans, emphasizing informed consent as a key component [1-4]. The development of digital documentation and therapy systems in health care since the late 20th century led to the availability of routine medical data. The era of big data in health care has since come with great promises to improve health care quality on individual and systemic levels [5,6] but also made crucial changes in the understanding of consenting into data and tissue use for scientific purposes necessary [7]. Accordingly, routine data from medical care are highly sensitive and require the same ethical considerations as other human research. Up to now, this type of secondary use of routine data requires individual patient consent if no other legal justification is applicable. This principle of study-specific consent is particularly challenging in health data science, where research questions may arise long after routine data are collected.

The storage, use, standardization, and exchange of medical data from multiple sources are prerequisites for interconnected research. Different approaches have been taken internationally to overcome barriers to using routine health data for research [8,9], but no established national systems have emerged yet. Recent policy impulses in Germany aim to simplify the use of data for research, as emphasized by the development of broad consent (BC) since 2018 or the Health Data Usage Act of 2024 [10], which are both in line with the General Data Protection Regulation [11].

The Medical Informatics Initiative [12], initiated by the German Federal Ministry of Education and Research, aims to reorganize, standardize, and simplify access to routine data. The concept of BC allows patients to participate in research outside a prespecified research project while complying with the principles of informed consent [13]. In this nationwide agreed framework, the BC allows the patients to provide their data in 4 categories (medical, insurance, and contact data as well as biomaterials). From the date of obtaining consent, the BC permits data extraction over a retrospective and prospective period of 5 years (allowing individual data extraction from a total 10-year interval). The storage and use of data from this interval is permitted for up to 30 years. Key steps for the establishment of the BC included compliance with different European federal and state data protection requirements as well as the implementation of interoperability standards [13-16].

Until now, data on the success rate and the perception of BC by patients in real-world care environments like emergency departments (EDs) are scarce [17-20]. Furthermore, it is unknown how potential selection bias is introduced when recruiting patients in the ED to consent to the BC. This is plausible as patients seeking emergency care face different challenges when being approached for research purposes than patients in elective care settings. These challenges range from impaired mental capacity, which may be the cause for—or an effect of—the urgent need for medical care, to limited time available for nonmedical procedures in the ED and the potential perception of research in the ED as a barrier to effective care.

Therefore, we aimed to establish a Medical Informatics Initiative–compliant BC process in the ED of the study site as a prototypical place to determine the primary success rate for obtaining BC depending on different modes of information about the BC. After reviewing the consented patients, we aimed to identify potential selection biases in consenting patients in the ED, where a large variety of patients with different chief complaints and diagnoses are treated. Variables of interest for determining potential selection bias include age, sex, triage category (urgency), admission and billing details (inpatient or outpatient), and presentation time. Finally, we aimed to identify which of the latter variables influenced the rate of dropout from the study and the rate of consent to BC.

## Methods

### Ethical Considerations

The study was approved by the local ethics committee of the University of Freiburg (22-1202-S1) and registered with the German Clinical Trials Register (DRKS00028753). Informed consent was obtained through a 2-step process. First, patients were provided with verbal information about the study and could consent to receive further information about BC. Second, detailed information about BC was provided, allowing patients to make an informed decision. Patients were free to opt in or opt out at any point during this 2-step approach. All analyses were performed on deidentified routine data collected and stored in the local electronic health records. Participants did not receive compensation for their participation in the study or consenting to BC. This publication does not include identifiable features that could reveal the identity of individual participants.

### Study Design

We conducted a single-center, prospective, observational study from September to December 2022 in a tertiary ED in Germany. The study was embedded in a multicenter study assessing BC in different consenting environments (NUM CODEX-Plus, DRKS00030054). The reporting of this study followed the STROBE (Strengthening the Reporting of Observational Studies in Epidemiology) statement for observational studies [21].

### Recruitment

We drew a sample of patients, selecting every 30th patient who presented to the ED and approached eligible patients. Eligible patients defined as >17 years of age, German-language proficiency (ability to read, speak, and understand German language as assessed by the recruiting staff), and mentally capable (absence of neurological impairment, eg, stroke, delirium, dementia, substance intoxication, or iatrogenic sedation at the time of presentation). We approached eligible patients in a 2-step consenting process. First, patients were asked if they were willing to participate in the BC study in general. Second, after giving verbal consent to the first step, patients were informed about BC and asked for written consent to any data category covered by BC.

The data categories, also referred to as BC modules, encompassed routine data (medical data generated during routine medical treatment), insurance data (data deposited with the health insurance company), biomaterials (tissue samples or body fluids), and contact data (to potentially recontact the patients for recruiting or information about incidental findings in the data analysis). Possible outcomes in this step were “consent” (if consent to at least 1 module was “yes”) or “no consent” (if the answer to all modules was “no”) or “dropout” (if no written response was received after verbally consenting or refusing the BC).

### Consenting

Participants could indicate their agreement or disagreement with each module independently. From the time of consent, a retrospective and prospective consent for 5 years could be selected by the participants, leading to a maximum period available for data extraction of 10 years. The use from the consented 10-year period was allowed for 30 years.

We applied three different modes of patient information and consenting: (1) direct in-person consenting in the ED, (2) delayed in-person consenting on the ward, or (3) delayed consenting via telephone after discharging home following the ED or inpatient stay. When consenting was performed in mode 3, we sent the consenting material to the participants by mail and awaited a written response. The mode of consent was determined by the random inclusion of the patient and the availability of the study nurses (eg, if a patient was included at 3 AM, a study nurse was notified upon starting their shift at 8 AM, and the patient was approached either in the ED, on the ward after admission, or by telephone if the patient had already been discharged). The working hours of the study nurses were 8 AM to 5 PM on weekdays. Consent was given digitally using a generic informed consent system [16] or in paper format. All consents were subsequently translated into Fast Healthcare Interoperability Resources for further use [14,15].

The primary outcome was the success rate of consenting to the BC within 30 days after presenting to the ED. We chose a 30-day interval, considering the interval between discharge from the ED or ward until contact could be made by telephone and the processing times of postal services (sending out and receiving consent documentation). The secondary outcomes were the diagnosis of the consented patients, the dropout rates, and the interaction duration needed to obtain consent. The interaction duration was defined as the time of discussion used for explaining the BC concept to the patients. It could be halted and continued at the request of the patient and ended with the decision on and written confirmation of consent or no consent.

### Analysis

To analyze the rates for the outcomes “consent,” “no consent,” and “dropout,” we calculated the proportions of each outcome per mode of information after excluding duplicates. The rate of dropouts was calculated in relation to all patients in the different information modes, whereas the rate for consent referred to the retained patients. The latter was defined as the patients with a recorded decision for or against the BC.

To evaluate possible selection bias between patients in the groups for consent, no consent, and the remaining ED population in the study period, we performed explorative descriptive statistics (ie, chi-square, Fisher exact test, and Wilcoxon rank sum tests). We analyzed the variables age, sex, triage category (Emergency Severity Index), billing details (inpatient or outpatient treatment), and on-hour presentation (ie, 8 AM through 5 PM). Differences in the *interaction duration* were analyzed using a Kruskal-Wallis test. Dunn comparison for multiple testing was used where applicable. To analyze the distribution of diagnoses in patients who did or did not consent, we performed a chi-square test after grouping the *International Classification of Diseases, Tenth Revision* (*ICD-10*) chapters to obtain a minimum of 5 diagnoses per group.

Further, we conducted two multiple logistic regressions to test for influence on the dependent variables (1) consenting to the BC (consent or no consent) and (2) on dropout of the study (dropout or no dropout). In the regression models, we included the independent variables age, sex, billing details, on-hour presentation, information mode on BC, and interaction duration. The information mode was, therefore, further categorized as either “in person” (information mode 1 and 2) versus “by telephone” (information mode 3) and as “direct information in the ED” (information mode 1) versus “delayed outreach” (information mode 2 and 3). Missing single data points were indicated in the analysis. Patients with incomplete datasets (ie, missing data for all variables named above) were excluded from the analysis. The software used for analyzing the data was GraphPad Prism (version 9.5.1; GraphPad Software, LLC).

## Results

### Recruitment

**Figure 1:**
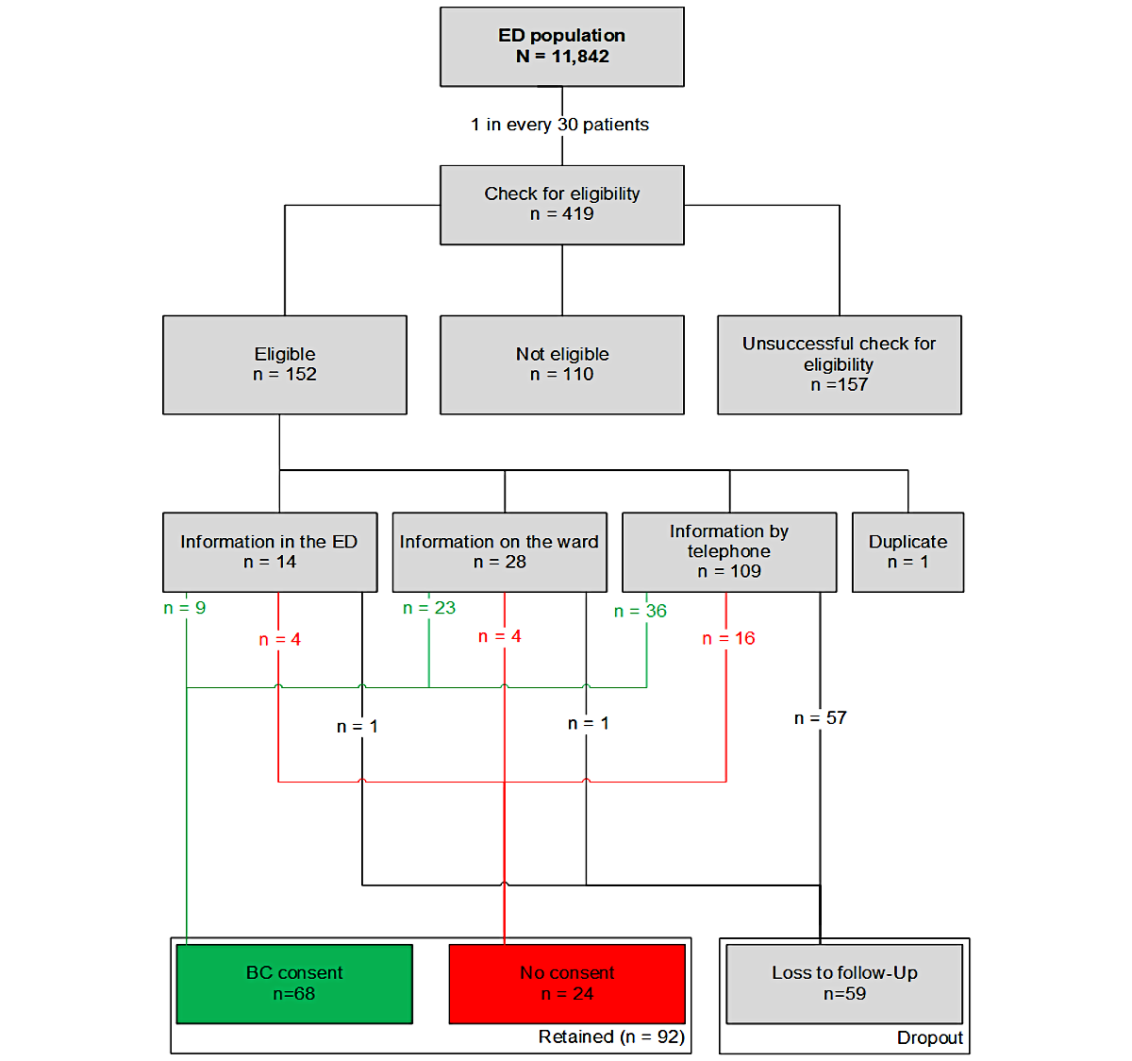
Flowchart of recruiting, informing, and consenting the ED population from the study period. BC: broad consent; ED: emergency department.

Within 86 days from September 19 to December 13, 2022, 11,842 patients presented to the ED of the study site. We randomly selected every 30th patient (n=419, 3.5%) who presented to the ED for eligibility to participate in the study (Figure 1). In total, 3 study nurses provided information and collected required consent forms; 1 performed all in-person information in the hospital, and 2 conducted required phone calls. All study nurses were female.

A total of 110 patients were not eligible for enrollment due to minor age (n=38), lack of sufficient German-language proficiency (n=24), mental impairment (dementia, stroke, and delirium; n=40), or death (n=7). The eligibility could not be assessed in 157 patients due to unsuccessful outreach attempts with the respective patients within 30 days after presenting to the ED (eg, incorrect contact information in the electronic health record data, or no response after initial contact, or could not be contacted during inpatient stay). A total of 151 patients were eligible for enrollment (excluding 1 duplicate patient) and further evaluated for potential consenting to BC.

We applied three modes of information about BC, that is, (1) in the ED on 14 (9.3%) patients, (2) during the consecutive inpatient treatment in the ward on 28 (18.5%) patients, or (3) by telephone after discharging home on 109 (72.2%) patients.

Of the 151 eligible patients, we did not receive the signed consent forms within 30 days after initially agreeing to participate from 59 (39.1%) patients (dropouts; Table 1). The dropout rates were 7.1% (n=1) in the ED information group and 3.6% (n=1) in the ward consenting group. Significantly higher rates were observed in the information by telephone group (n=57, 52.3%; *P*<.001).

**Table 1:** Dropout rates of the 151 eligible patients, depending on the mode of information about the broad consent (BC).

Mode of information about BC	Retained		Dropout		*P* value
	Values, n (% of eligible patients)	Values, n (% within information mode)	Values, n (% of eligible patients)	Values, n (% within information mode)	
ED^a^	13 (8.6)	13 (92.9)	1 (0.7)	1 (7.1)	<.001^b^
Ward^c^	27 (17.9)	27 (96.4)	1 (0.7)	1 (3.6)	<.001^b^
Telephone^d^	52 (34.4)	52 (47.7)	57 (37.8)	57 (52.3)	<.001^b^
Total	92 (60.9)	—^e^	59 (39.1)	—	—

^a^In the emergency department (ED) on 14 patients.

^b^Chi-square test.

^c^During the consecutive inpatient treatment in the ward (n=28).

^d^By telephone after discharging home (n=109).

^e^Not applicable.

### Consenting

Of the 151 eligible patients, consent to at least 1 module of the BC was given by 68 (45%) patients, while 24 did not consent by either refusing the information about BC (n=19, 12.6%) or all the modules on the consent form (n=5, 3.3%). No significant differences between the 3 modes of information were detected when comparing the given consents and the not-given consents (*P*=.28; Table 2). One patient was drawn twice into the random sample (ie, in consecutive ED visits) and handled as a duplicate. Thus, we recorded the responses of 92 patients (60.9% of eligible patients) within the study period.

To check if the eligible patients significantly differ from the ED population, we tested for differences in the variables age, sex, triage category, inpatient status, and on-hour presentation. Of the 11,842 patient records, 104 patient records were missing data for all variables mentioned earlier and were hence excluded from the ED population. The 152 eligible patients from the sample were not significantly different from the residual ED population (n=11,586; Table 3).

We applied the same comparison between the 68 patients who consented to at least 1 module of the BC and the 24 who did not. None of the analyzed variables were significantly different between the 2 groups (Table 4).

The interaction duration needed to inform the patients on the BC differed between the modes of information. The mean duration was 0.53 (95% CI 0.39-0.67) hours in the ED group and 0.53 (95% CI 0.47-0.60) hours in the ward group (*P*>.99). The time used to inform patients in the telephone group was significantly shorter, with 0.15 (95% CI 0.13-0.16) hours (*P*<.001 in both comparisons).

**Table 2:** Retained responses (n=92) to the broad consent (BC) information, depending on the mode of information^a^.

Mode of information about BC	Given consent		No consent			*P* value
	Values, n (% of retained patients)	Values, n (% within information mode)	Values, n (% of retained patients)	Values, n (% within information mode)		
ED^b^	9 (9.8)	9 (69.2)	4 (4.4)	4 (30.8)	.28^c^	
Ward	23 (25)	23 (85.2)	4 (4.4)	4 (14.8)	.28^c^	
Telephone	36 (39.1)	36 (69.2)	16 (17.4)	16 (30.8)	.28^c^	
Total	68 (73.9)	—^d^	24 (26.1)	—	—	

^a^Consent was given by 68 patients, and 24 patients did not consent.

^b^ED: emergency department.

^c^Chi-square test.

^d^Not applicable.

**Table 3:** Descriptive statistics of patients eligible for consenting to the broad consent according to the study protocol and of the residual emergency department (ED) population during the study period.

	Eligible patients from random sample (n=151)	ED population (n=11,586)	*P* value
Age (years), median (IQR)	47.0 (31.0-68.0)^a^	47.0 (27.0-68.0)^b^	.41^c^
Sex (female), n (%)	75 (50)^b^	5192 (44.8)^d^	.22^e^
Urgent triage category (Emergency Severity Index 1-3), n (%)	92 (60.9)^f^	7055 (60.9)^g^	.86^e^
Inpatient status, n (%)	52 (34.4)^b^	3541 (30.6)^h^	.29^e^
On-hour presentation, n (%)	58 (38.4)^a^	4422 (38.2)^a^	>.99^e^

^a^Missing n=0.

^b^Missing n=1.

^c^Mann-Whitney *U* test.

^d^Missing n=4.

^e^Fisher exact test.

^f^Missing n=6.

^g^Missing n=268.

^h^Missing n=3.

**Table 4:** Descriptive statistics of the 68 patients who consented to the broad consent and the 24 who did not.

	Given consent (n=68)	No consent (n=24)	*P* value
Age (years), median (IQR)	47.5 (30.8-68.8)^a^	55.5 (34.5-72.8)^a^	.41^b^
Sex (female), n (%)	26 (38.2)^a^	13 (54.2)^a^	.23^c^
Urgent triage category (Emergency Severity Index 1-3), n (%)	42 (61.8)^d^	15 (62.5)^e^	>.99^c^
Inpatient treatment, n (%)	31 (45.6)^a^	8 (33.3)^a^	.34^c^
On-hour presentation, n (%)	29 (42.7)^a^	10 (41.7)^a^	>.99^c^

^a^Missing n=0.

^b^Mann-Whitney *U* test.

^c^Fisher exact test.

^d^Missing n=3.

^e^Missing n=1.

### Distribution of Diagnoses

To determine the extent to which our research included a representative sample of diagnoses, we conducted a comparative analysis of the recorded diagnoses of patients who consented to the BC and those of the ED population in the study period. The diagnoses were classified according to the chapters of the *ICD-10* catalog. If multiple diagnoses were entered for a single patient, all diagnoses were included in the statistical analysis with equal weight. A chi-square test indicated that there was no statistically significant difference between the 2 groups with a *P* value of .49 (Table 5).

**Table 5:** Comparison of the diagnoses of the patients who consented to the broad consent and the remaining emergency department (ED) population^a^.

*ICD-10*^b^ chapter	Given consent (n=102), n (%)	ED population (n=16,594), n (%)	*P* value^c^
I-VIII	7 (6.9)	2056 (12.4)	.49
IX-X	10 (9.8)	1687 (10.2)	.49
XI-XVII	11 (10.8)	2080 (12.5)	.49
XVIII	24 (23.5)	3381 (20.4)	.49
XIX	21 (20.6)	3535 (21.3)	.49
XX-XXII	29 (28.4)	3855 (23.2)	.49

^a^If multiple diagnoses were available for a single patient in the electronic health record, we included all diagnoses in the statistical analysis with equal weight.

^b^ICD-10: International Classification of Diseases, Tenth Revision.

^c^Chi-square test.

### Factors Influencing the Success Rate of Consenting to BC

We performed multiple logistic regressions to identify relevant influences on the consent rate. The variables included age, sex, inpatient status, on-hour presentation, information mode, and interaction duration. A longer interaction time (the duration of the information about the BC) resulted in a significantly higher approval rate (Table 6). None of the other variables tested significantly affected the approval rate.

**Table 6:** Multiple logistic regression on factors influencing the consent rate to broad consent of the 92 retained patients^a^.

Variable	Odds ratio (95% CI)	|*Z*|	*P* value
Age (years)	0.98 (0.96-1.01)	1.32	.19
Sex (female)	0.34 (0.11-0.98)	1.96	.05
Inpatient treatment	1.37 (0.34-6.12)	0.44	.66
On-hour presentation	1.54 (0.53-4.74)	0.78	.44
Face-to-face information	0.49 (0.05-4.53)	0.63	.53
Information during emergency department stay	0.20 (0.03-1.36)	1.64	.10
Interaction time (hours)	*130.80 (2.14-19,119.51)^b^*	*2.13*	*.03*

^a^Continuous variables are given with the respective unit.

^b^Significant results are displayed in italics format.

### Factors Influencing the Dropout Rate

In the second step, we used multiple logistic regression to ascertain possible influences on the dropout rate (Table 7). Face-to-face information was found to be associated with significantly lower odds of dropout. Female sex, however, increased the odds ratio for dropout. No other variables tested demonstrated a significant effect on the dropout rate.

**Table 7:** Influencing factors on the dropout rate (dependent variable) of 150 patients, calculated by multiple logistic regression^a^.

Variable	Odds ratio (95% CI) (profile likelihood)	|*Z*|	*P* value
Age (years)	1.00 (0.98-1.02)	0.30	.77
Sex (female)	*2.51 (1.15-5.63)^b^*	*2.28*	*.02*
Inpatient treatment	1.67 (0.61-4.81)	0.98	.33
On-hour presentation	0.58 (0.26-1.30)	1.31	.19
Face-to-face information	*0.07 (0.003-0.63)*	*2.10*	*.04*
Information during emergency department stay	0.24 (0.01-6.81)	0.95	.34
Interaction time (hours)	0.04 (0.001-2.43)	1.49	.14

^a^Continuous variables are given with the respective unit.

^b^Significant results are displayed in italics format.

## Discussion

### Principal Findings

Our study was conducted in a tertiary ED in Germany and aimed to assess the primary success rate for obtaining BC among ED patients. Second, we aimed to identify potential selection biases and to identify factors influencing the rate of consent and dropout. ED patients typically present with a broad spectrum of diseases and may stem from different cultural and lingual backgrounds. In addition, patients in the ED are often discharged home after the ED treatment. We therefore deemed the ED an ideal environment for testing the BC, as it reflects a broad spectrum of complaints and diseases rather than preselected conditions. Therefore, the availability of ED data by BC for research might yield insights into system-wide health care service delivery environments.

Of 11,842 patients, 419 were screened for eligibility according to the protocol. Of these, 151 patients were eligible for the study. Among the eligible patients, 68 (45%) consented to at least 1 BC module, while 19 (12.6%) refused participation. In total, 59 (39.1%) patients gave verbal consent by telephone but did not return the signed consent forms sent by mail after the call within 30 days (ie, dropout). Patient information about BC was performed in 3 different modes. The highest consent rates observed when informing patients face-to-face on the ward following the ED stay (23/27, 85.2%), while those approached in the ED or by telephone tended to be less likely to consent (9/13, 69.2%), although no significant difference was detected. Dropout rates were highest in telephone-based information (57/109, 52.3%). The different success rates in the 3 modes of information yield questions regarding the comprehensible presentation of information via telephone, the possible interindividual differences between the study nurses, and potential influences on perceived patient autonomy during episodes of illness requiring inpatient care [22-24]. In addition, in our data, female sex was associated with higher dropout rates. While the latter is documented in the literature already, it emphasizes concerns regarding sex bias in medical research [25,26]. Yet, no significant differences were found between the consenting and nonconsenting groups concerning age, triage category, billing detail (inpatient status), presentation time, or the included diagnoses. Therefore, we deem selection bias beyond the above to be unlikely.

### Limitations

The study faced several limitations. First, the inclusion of a relatively small sample size due to a high rate of primarily ineligible patients, together with the exclusion of non-German speakers, may have introduced selection bias in these patients who, nonetheless, represent a substantial subset of patients in the ED. Furthermore, the single-center design in a German tertiary-level academic hospital limits the generalizability of our findings.

Second, the number of patients drawn into the sample who could not be checked for eligibility led to a decreased number of retained patients, which likely reduces the generalizability of our findings. The single-center design may additionally limit the generalizability.

Third, we see a high dropout rate in the group receiving information by telephone. As the patients educated by telephone were mostly patients who were priorly discharged home from the ED, this might introduce a potential bias in recruiting ambulatory or outpatients for BC. Fourth, identifying the interaction duration as a significant factor influencing the consent rate, another limitation stems from the missing distinction of the time needed to provide verbal information and the time needed to obtain written consent by the patient. Although the mere documentation of the signature was not found to be a major obstacle in the consenting process by the study personnel, we cannot exclude an effect of this measurement uncertainty. A longer interaction duration in consented patients could therefore partly stem from the additional time needed to obtain the signature of the patient.

Finally, although no significant differences were found in the distribution of urgent or nonurgent patients in analyzed groups, details of patients who were severely ill or injured may remain undetected due to their small proportion in relation to the overall ED turnover.

### Comparison With Prior Work

Previous studies have highlighted the challenges of obtaining informed consent in preselected patient cohorts (eg, oncologic care) [16-18,20]. Our study adds to the existing literature by providing specific data on real-life consenting rates in a tertiary ED. In total, 45% (n=68) of the eligible patients in our study consented to at least 1 BC module in a real-world care setting compared to simulation-based consenting scenarios of 86.9% in a study by the German Center for Inflammation Medicine [17] or 78.8% in another simulation-based German-Dutch consent scenario [18]. For the first time, this study evaluated the effectiveness of different consenting modalities and therefore provides realistic recommendations for the use of BC in ED environments. Rather than assessing patient understanding and motivation regarding the BC [17,18], our work demonstrates that consenting patients in the ED to BC yields a widely representative sample of the ED population.

### Practical Implications

Although conducted at a single German site, our research yields the general finding that obtaining BC in ED is a challenge that requires tailored information about BC for specific patient populations. As the most successful mode of information was face-to-face on the ward following the ED stay, this approach seems to be the most promising for including inpatients. Yet, efforts to explore and strengthen patient autonomy need to be taken. For the consenting of discharged patients, a more sophisticated approach is needed. Either these patients have to be informed during the ED stay, which implies a 24/7 availability of study personnel, or the telephone-based information of this cohort needs to cover a more structured or at least longer recall interval to ensure the verbally given consent is documented in the returned consent material. Furthermore, web-based information material could improve this process. In addition, given the high rate of primarily ineligible patients, future strategies should focus on overcoming language barriers and implementing protocols for the inclusion of patients who are unable to consent by themselves (eg, those who are minors, mentally impaired, and deceased). To establish population-wide applicability, measures to support the retention of female patients need to be considered.

### Conclusions

The study demonstrates that obtaining BC in an ED population is feasible, with an overall consent rate of 45% (68/151) among eligible patients across all information modes tested. However, consenting on the ward following the ED stay showed the highest success rate with 85.2% (23/27). No selection bias was found, but higher dropout rates among female patients and patients who had already been discharged home were observed. The patients who had been primarily ineligible to consent due to a lack of German-language proficiency or other causes (eg, those who are minors, mentally impaired, and deceased) underscore the need for improving consenting efforts by providing multilingual BC information and by introducing consent options for next of kin for patients. Whether more sex-specific information is needed should be evaluated by further research. Future research should also validate the reported findings in multicenter approaches and focus on reducing potential residual biases.

## Abbreviations

BC: broad consent
ED: emergency department
ICD-10: International Classification of Diseases, Tenth Revision
STROBE: Strengthening the Reporting of Observational Studies in Epidemiology
